# The Geriatric Forensic Psychiatry Rotation at University of Chicago: Utilization and Educational Benefit of a Subspecialty Rotation in Psychiatric Residency Training

**DOI:** 10.3389/fpsyg.2019.02123

**Published:** 2019-10-02

**Authors:** Carolyn Shima, Sanford Finkel, Deborah Spitz, Amanda I. Goldstein

**Affiliations:** ^1^The University of Chicago, Chicago, IL, United States; ^2^University of Chicago Medicine, Chicago, IL, United States; ^3^University of California, Los Angeles, Los Angeles, CA, United States

**Keywords:** testamentary capacity, undue influence, forensic psychiatry, training, geriatric psychiatry and aging, psychiatry training programs

## Abstract

Ten years ago, the University of Chicago Department of Psychiatry and Behavioral Neuroscience established a geriatric forensic psychiatry rotation for 4th-year psychiatry residents as one option in a required experience in forensic psychiatry. The rotation’s primary emphasis was to learn about cases of testamentary capacity and undue influence. The program was supervised by a senior faculty member, an experienced clinician who was board-certified in Geriatric Psychiatry.

**Results:**

Over the past decade, three of the 11 residents have pursued career paths in forensic psychiatry, while another has become a geriatric psychiatrist. More than half of the respondents have pursued geriatric and/or forensic work following their graduations, and all believe that what they learned in the rotation applied to their general practice work. All 11 indicated that the rotation increased their interest in and understanding of forensic work. Ten “strongly agreed” that the geriatric forensic psychiatry rotation was a valuable learning experience; one “agreed.” On average, trainees worked on 4.64 forensic cases over the course of the rotation and attended 2–3 trials or depositions. Over the last 3 years of the program, all three of the participating residents have chosen to complete a forensic fellowship following the rotation.

**Conclusion:**

Residents affirm that a geriatric forensic psychiatry rotation is a valuable learning experience, one that has utility after their graduation. The University of Chicago Department of Psychiatry and Behavioral Neuroscience is committed to continuing this rotation as an important part of their forensic experience in resident education and to encourage more interest in the area of geriatric psychiatry.

## Introduction

There are many specialized clinical and educational experiences offered within psychiatry residency programs in the United States and Canada, the content of which may depend in part on departmental expertise, priorities, and orientation, as well as on geography and resident interest. Some of these educational experiences are well established, while others are relatively new; some involve all residents in a program, while others are elective and appeal to only some residents each year; some have been studied, while others, unstudied, remain important experiences within residency programs. A handful of such specialized programs are presented each year at the annual meeting of AADPRT, the American Association of Directors of Psychiatric Residency Training, and serve as models for similar programs at other sites. The University of Chicago has crafted one such training program, focused on geriatric forensic psychiatry. In our review of the literature, we did not find any examples of similar studied rotations focusing on the intersection between geriatric and forensic psychiatry.

Over the last 10 years, the American Board of Psychiatry and Neurology reports issuing 17,189 board certifications for the specialty of Psychiatry. Of those, there were 694 board certifications for forensic psychiatry and 733 for geriatric psychiatry. Forensic and geriatric psychiatry continue to be less represented in residency education in specialized training experiences in comparison to the more common rotations for residents of consult-liaison psychiatry, child and adolescent psychiatry, and addictions psychiatry. The University of Chicago, like many programs, provides residents with the opportunity to observe, assist, or write a shadow report on at least one forensic case during their residency training. At many institutions, this is the only forensic opportunity available to residents. The University of Chicago has crafted a more robust training experience to allow interested residents a hands-on, one-on-one training experience focused specifically in these two frequently overlooked divisions of psychiatry.

Ten years ago, the University of Chicago Department of Psychiatry and Behavioral Neuroscience established a specialized training experience to increase resident knowledge and experience in geriatric and forensic psychiatry. The rotation has never been formally assessed as to its impact on learning or residency experience. The rotation’s primary emphasis was on exposing residents to cases of testamentary capacity and undue influence in geriatric forensic psychiatry. The program was supervised by a senior faculty member, an experienced psychiatrist with over 30 years experience as an expert witness, and national recognition in the area of geriatric forensics.

The intersection of geriatric and forensic psychiatry occurs most frequently in cases of testamentary capacity in which a person’s ability to knowingly and purposefully execute a will comes into question. Challenges to executed wills occur most commonly after the testator has expired and so the majority of testamentary capacity cases occur when a personal evaluation of the testator is no longer possible. As a result, particular expertise in the evaluation of medical records, evaluation of suspicious circumstances surrounding a will, and evaluation of family dynamics becomes particularly important. When the validity of a will is in question, many testamentary capacity cases also raise the concern for undue influence, or an allegation that a beneficiary has exerted coercive or excessive influence to override the testator’s wishes in executing a will. Our senior faculty member focuses on testamentary capacity and undue influence cases in his work, and so the majority of the geriatric forensic rotation consists of residents observing and assisting in these two challenging yet common case topics.

Residents rotated over the course of an entire year in their PGY-4 training year. During the year, residents met with the senior faculty member at least weekly to discuss and participate in his ongoing gero-forensic case-work. Residents discussed geriatric forensic cases and issues, reviewed methodology and literature, assisted with deposition preparation and research, and were given the opportunity to participate in attorney meetings regarding legal cases in which the faculty member was engaged as an expert witness. Residents were also given the opportunity to travel to observe depositions and expert witness testimony at trial on the cases they followed.

Forensic topics of discussion and learning included forensic report writing, expert witness testimony, biological/psychiatric/legal determinants of testamentary capacity, biological/psychiatric/legal determinants of undue influence, evaluation of family dynamics and relationships, ethics in forensic psychiatry, private practice management, retainer agreements, and managing attorney expectations. Geriatric topics of discussion and learning included neuro-cognitive disorders and dementias, cognitive testing methods, evaluating medical records, health conditions specific to the geriatric population, pharmacological management in geriatric patients, and psychosis/delirium in the geriatric population.

In this study, we aimed to measure the utilization of the rotation, experience of residents, and educational benefit to residents who completed the year-long elective specialized training experience.

## Methods

Graduates of the University of Chicago Psychiatry Residency Program who had completed the Geriatric Forensic Psychiatry Rotation between 2011 and 2019 were invited by email to fill out a survey regarding their experiences on the rotation, educational benefit, and attitudes toward the geriatric forensic psychiatry rotation. The survey was completed by 11 of the 12 graduates. Responses were voluntary and anonymous. Written informed consent was obtained from participants. The survey was designed using REDCap and consisted of 30 questions. Question format consisted of Likert scales, slider questions, forced-choice (yes/no) responses, numerical responses, and narrative comments. Information obtained included both qualitative and quantitative data regarding the educational experience as well as details of post-graduation geriatric and forensic psychiatry practice.

Ten Likert scale questions were scored on a scale of strongly agree, agree, disagree, or strongly disagree. They consisted of the following:

1. I will be likely to pursue forensic work in the future, or I have pursued forensic work since completing the rotation.
2. The geriatric forensic psychiatry rotation gave me a better understanding of forensic psychiatry.
3. The geriatric forensic psychiatry rotation gave me a better understanding of geriatric psychiatry.
4. I feel knowledgeable in the topic of undue influence.
5. I feel knowledgeable in the topic of testamentary capacity.
6. I feel knowledgeable in the topic of insane delusions.
7. I feel the geriatric forensic psychiatry rotation was a valuable learning experience.
8. The geriatric forensic psychiatry rotation increased my interest in forensic psychiatry.
9. The geriatric forensic psychiatry rotation provided me with a clear understanding of expert witness testimony.
10. The geriatric forensic psychiatry rotation educated me about court procedure.

Participants were then asked to answer five slider scale questions ranking their knowledge prior to and following the rotation on a scale from 0 to 100. The questions consisted of the following:

1. How knowledgeable did you feel about forensic psychiatry prior to the rotation?
2. How knowledgeable did you feel about forensic psychiatry after the rotation?
3. How knowledgeable did you feel about geriatric psychiatry prior to the rotation?
4. How knowledgeable did you feel about geriatric psychiatry after the rotation?
5. [If a fellowship was pursued after the rotation] How prepared did you feel by the geriatric forensic rotation?

Participants were then asked to provide a numerical response to the following questions. A space for comments was provided.

1. How many depositions did you observe over the course of your rotation?
2. How many cases did you work on over the course of your rotation?
3. How many instances of expert witness testimony did you observe at trial or deposition?
4. How many meetings with attorneys did you participate in over the course of your rotation?
5. How often did you meet with the supervising faculty?
6. Approximately how many hours/week did you dedicate to this rotation?

Finally, participants were asked to answer yes/no questions with a space for comments, and a few short answer questions.

1. Have you pursued forensic or geriatric work in your career?
2. Since graduating, have you worked with geriatric patients?
3. Since graduating, have you taken on any forensic cases?
4. Did you do a fellowship in geriatrics or forensics?
5. Was (the time spent per week on the rotation) sufficient to have a full experience?
6. Are there things you learned that apply to your general practice work? Please explain.
7. Are there any specific vignettes or information that stand out for you? Please explain.
8. What was your favorite part of the experience?
9. What was your least favorite part of the experience?

## Results

The 11 graduates dedicated an average of 4.09 h per week to the Geriatric Forensic Psychiatry Rotation. Nine participants (82%) met with the senior faculty member at least once a week throughout the year-long rotation. On average, residents focused on 5.00 forensic cases (range 2–12) over the year. Along with the senior faculty member, residents participated in an average of 4.64 (range 1–14) attorney meetings. The average number of occurrences of expert witness testimony observed at depositions and/or trials was 2.64 (range 1–6).

On a scale from 0 to 100, where 0 indicated “not knowledgeable at all” and 100 indicated “extremely knowledgeable,” participants felt their knowledge about forensic psychiatry increased by 44.28 points on the scale after the rotation (Table 1). Similarly, participants felt their knowledge about geriatric psychiatry increased by 31.63 points on the scale after the rotation. The majority of graduates (72%) felt they knew less about forensics than geriatrics prior to the rotation.

**TABLE 1:** Knowledge of forensic and geriatric psychiatry on a 100-point scale before and after the rotation.

	**Knowledge of forensic psychiatry**	**Knowledge of geriatric psychiatry**
Prior to the rotation	31.27	40.55
After the rotation	75.55	72.18
Knowledge gained	44.28	31.63

The 11 graduates were unanimous that the rotation provided education on court procedure and an understanding of expert witness testimony. Ten graduates (91%) felt knowledgeable on the topics of testamentary capacity and insane delusions at the time of the survey. Nine graduates (82%) felt knowledgeable on the topic of undue influence at the time of the survey. Eight participants “strongly agreed” and three “agreed” that the rotation increased their interest in forensic psychiatry. Ten participants “strongly agreed” and one “agreed” that the rotation was a valuable learning experience.

**FIGURE 1:**
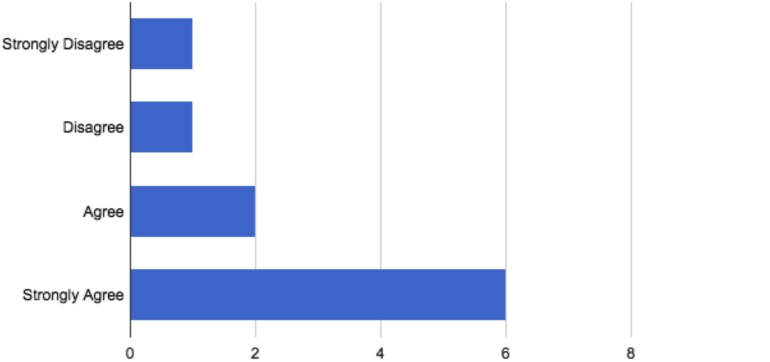
Participant response of whether they are likely to pursue/have pursued forensic work following the rotation.

Eight participants “agreed” or “strongly agreed” that they are likely to pursue or have already pursued forensic work following graduation (Figure 1). Three of the 11 participants (27%) will complete or have completed a fellowship in forensic psychiatry by 2020. Eight graduates (72%) have worked with geriatric patients, and one of these graduates specializes in geriatric psychiatry. All 11 participants (100%) believed that what they learned during the rotation was applicable to their general practice work.

Over the last 3 years of the program, all three of the participating residents have chosen to complete a forensic fellowship following the rotation. One prior graduate reported that they did not complete a forensic fellowship, but actively work on forensic cases in their current practice. Seven of the eleven participants indicated that they have worked with geriatric patients since their graduation, and one reported they planned to after their graduation. One participant commented that they had more exposure to civil forensic work during the rotation than they had during their entire fellowship year. Participants also reported specific learning which has been useful in their general practice, such as knowledge about “cognitive screening,” “teamwork,” “the writing of concise clinical progress notes to improve patient care,” and psychiatric “ethics.” One participant wrote that due to the rotation, “The knowledge of ways in which elderly can be taken advantage of and what to watch out for is something that is on my mind with patients daily.” There were no negative comments regarding the content or experience of the rotation.

## Discussion

In our examination of the utilization, experience of residents, and educational benefit of this specialized training experience in geriatric forensic psychiatry, we found resident response to be overwhelmingly positive. The fact that at least one fourth-year psychiatric resident has chosen this elective experience each year underscores the popularity and worthwhileness of this rotation. Particularly interesting is the recent increase in participants choosing careers and fellowships in forensic psychiatry in the most recent 3 years. While the overall content and supervision approach has not changed much over the last decade, we hypothesize that word-of-mouth from senior to junior residents has increased the appeal of this rotation for residents contemplating forensic careers. It has also become more common for residents to seek fellowship training in psychiatry over the last 10 years nationwide, and forensic psychiatry has been gaining popularity in sub-specialty choice. The comments and responses from residents were highly favorable, and there was not a single negative comment regarding the content or benefit of the rotation. We would like to recognize the limitations of our study, particularly that some residents were asked to recall experiences that occurred many years ago, introducing recall bias to our conclusions. For the future, it may be helpful to provide this survey immediately following the rotation, and to provide additional questions regarding what lead to a choice of forensic or geriatric work in future practice.

To further characterize the types of cases and learning which occurred during this valuable rotation, we will discuss some of the main topics covered in education and some examples of the case-work. The following represent some of the cases and issues that have been highlighted in this rotation.

From a *biological* perspective, there are a range of medical conditions that are not commonly seen but have relevance to the issues of testamentary capacity and/or undue influence. A few examples:

1. The case of multisystem atrophy, an illness with profound physical effects, but with few cognitive deficits. Some of the children in the case asserted that their father lacked capacity and was unduly influenced. Discovery demonstrated father’s intact capacity and lack of susceptibility to undue influence, and the case settled favorably to the defense.

2. The case of a patient with Castleman’s disease, which led to severe fatigue, weight loss, and debilitation. The jury ruled that this woman was competent to execute a will but was vulnerable to undue influence.
3. The case of a 98-year-old woman who changed her will after a TIA. The judge ruled that she had the capacity to do so.

Other cases include mesothelioma, progressive supranuclear palsy, cancer with metastasis to the brain, and corticobasal degeneration. Further, multiple cases involve patients with delirium (Liptzin et al., 2010) or deathbed wills (Peisah et al., 2014).

Other *psychiatric and neurological* conditions:

1. A range of dementing illnesses (Major Neurocognitive Disorders) (DSM-5, 2013), which include Alzheimer’s disease, Frontotemporal dementia, Lewy Body dementia, Posterior Cerebral Atrophy, Traumatic Brain Injury, and Parkinson’s disease.

2. The case of a man with a diagnosis of schizophrenia, paranoid type, who believed that God was ordering him to poison the drinking water for his cattle so that they would die, but who otherwise fulfilled the criteria for possessing testamentary capacity.
3. The case of a severely depressed 89-year-old man, fearful of losing his caregiver, and thereby agreeing to leave her his estate.

Inasmuch as expert work generally requires arriving at a diagnosis, it is important to note that the DSM-5 must be interpreted broadly. For example, in a narrow sense, an illness such as Frontotemporal dementia requires cognitive testing and neuroimaging, even if the patient presents with advanced symptomatology, and clinical judgment is sufficient to establish a diagnosis. In such a situation, it is important to note that the DSM-5, more generally, does allow for clinical expertise to transcend the specific listed criteria.

*Psychological and psychosocial* considerations:

1. The most common litigation posits siblings in which one or more are provided a larger portion of the parent’s estate.
2. The second most frequently experienced litigation involves second (or third) spouses against children of prior marriages.
3. Indicia of undue influence are common in the elderly, especially sequestration and dependency (Peisah et al., 2009), and so a close examination of the indicia and how these fit into the larger picture of the medical records and family dynamics becomes important.
4. Another frequent element is the interpretation of neuropsychological testing: for many elderly, particularly those with medical illnesses, the multi-hour testing results in gradually declining performance. Moreover, many of the tasks are unfamiliar and may result in poor results because of lack of motivation. Thus, conclusions are best interpreted in the context of the person’s medical and psychiatric condition and motivation.

*Medico-legal* considerations:

Although courts generally allow experts to opine on susceptibility to undue influence, there is a lack of judicial consensus as to whether the expert can provide an opinion on undue influence. During this 10-year experience, on two occasions, courts in one state allowed the expert to testify on the ultimate question of undue influence, whereas subsequently a third court did not.

Older people often are exposed to painful “arrows,” such as illnesses, loss of spouses and friends, and financial setbacks. These are internal arrows and often unavoidable. As clinicians, we are repeatedly called upon to address these problems and help our patients. Undue influence represents an external arrow in which a perpetrator creates a situation which results in pain and suffering for the individual. As such, the problem is an extension of the clinical problems which we encounter on a regular basis.

## Conclusion

The geriatric forensic psychiatry rotation at the University of Chicago is a unique experience. While many other programs have developed niche experiences for residents to learn about particular areas of psychiatric practice, we did not find any published evidence that a similar program exists at other institutions. Universities tend to create these experiences in areas in which they have an especially robust clinical focus or faculty with particular expertise.

The results of the survey demonstrate that participants found the geriatric forensic psychiatry rotation to be successful in its aims of educating residents on the interface of forensic psychiatry and geriatric psychiatry, and the related issues of testamentary capacity and undue influence; giving them experience in these fields and interesting them in related careers.

## Data Availability Statement

The datasets generated for this study are available on request to the corresponding author.

## Author Contributions

CS provided coordination, contributed to the Introduction, Methods, Discussion, Conclusion, and Results sections, and managed survey administration and creation of survey. SF contributed to the survey development, wrote the Discussion section, and contributed to the editing. DS wrote the Introduction section, contributed to the survey participant/survey creation, and editing. AG assisted with survey creation/administration/dissemination, contributed to the Results and Methods sections, and editing.

## Conflict of Interest

The authors declare that the research was conducted in the absence of any commercial or financial relationships that could be construed as a potential conflict of interest.
